# Supplementary material for: Association between oral microbiome and seven types of cancers in East Asian population: a two-sample Mendelian randomization analysis
Source: Front Mol Biosci. 2023 Nov 21;10:1327893. doi: 10.3389/fmolb.2023.1327893 (PMC10702768; doi:10.3389/fmolb.2023.1327893)
Supplement: Supplementary file 1 [file DataSheet1.ZIP › Supplementary Materials/MR plots of saliva/saliva═╝/Breast cancer/pheno.2481_to_breast cancer_leave_one_out.pdf]

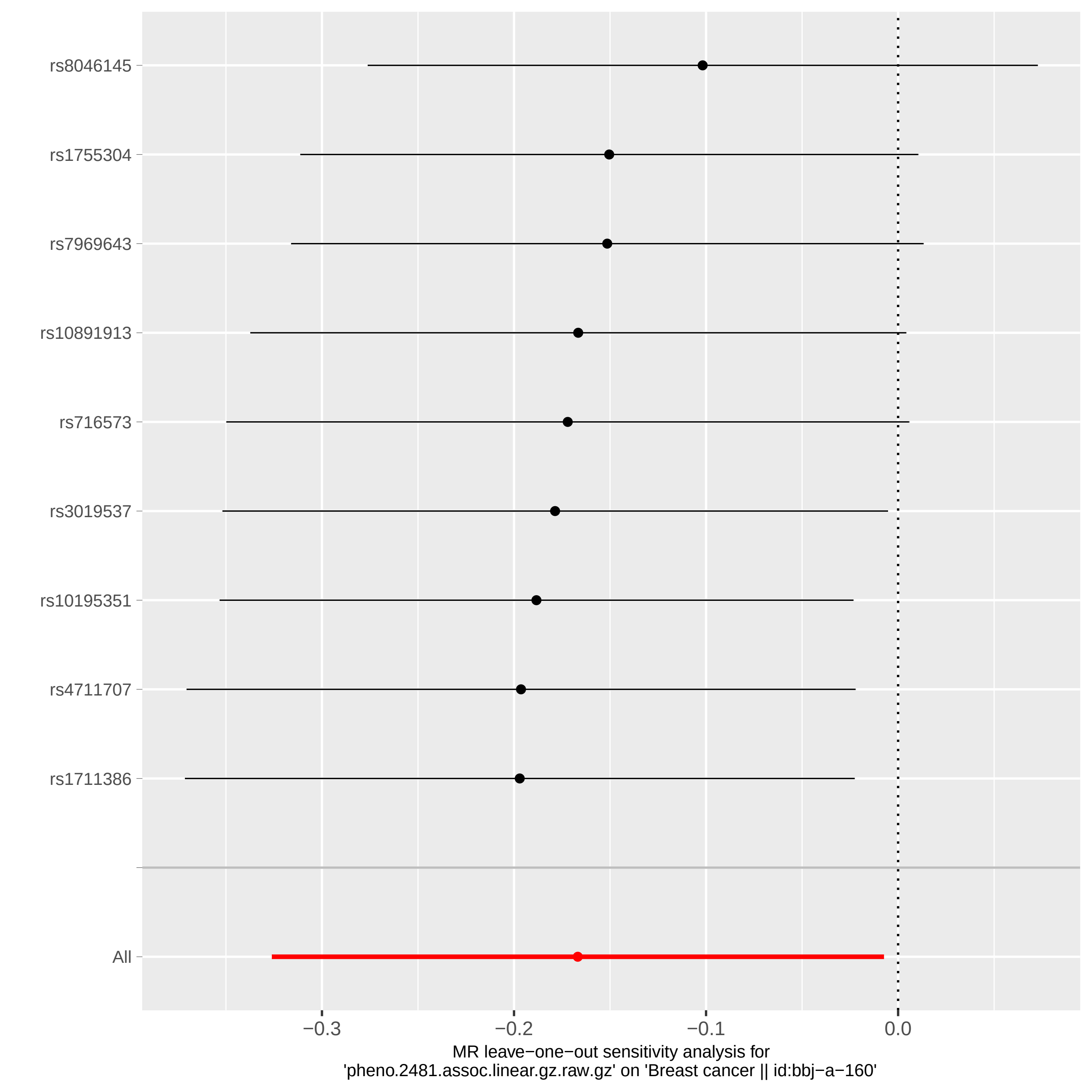
rs8046145
rs1755304
rs7969643
rs10891913
rs716573
rs3019537
rs10195351
rs4711707
rs1711386
All
−0.3
−0.2
−0.1
0.0
MR leave−one−out sensitivity analysis for
'pheno.2481.assoc.linear.gz.raw.gz' on 'Breast cancer || id:bbj−a−160'
